# Electrostatic-Force-Assisted Dispensing Printing to Construct High-Aspect-Ratio of 0.79 Electrodes on a Textured Surface with Improved Adhesion and Contact Resistivity

**DOI:** 10.1038/srep16704

**Published:** 2015-11-18

**Authors:** Dong-Youn Shin, Sung-Soo Yoo, Hee-eun Song, Hyowon Tak, Doyoung Byun

**Affiliations:** 1 Department of Graphic Arts Information Engineering, Pukyong National University, 365, Sinseon-ro, Nam-gu, Busan, 608-739, Republic of Korea; 2 Solar Energy Research Centre, Korea Institute of Energy Research, Daejeon, 305-343, Republic of Korea; 3 Enjet Inc., Ltd., 2066, Seobu-ro, Jangan-gu, Suwon-si, Gyeonggi-do, 440-746, Republic of Korea; 4 Department of Mechanical Engineering, Sungkyunkwan University, 2066, Seobu-ro, Jangan-gu, Suwon-si, Gyeonggi-do,440-746, Republic of Korea

## Abstract

As a novel route to construct fine and abnormally high-aspect-ratio electrodes with excellent adhesion and reduced contact resistivity on a textured surface, an electrostatic-force-assisted dispensing printing technique is reported and compared with conventional dispensing and electrohydrodynamic jet printing techniques. The electrostatic force applied between a silver paste and the textured surface of a crystalline silicon solar cell wafer significantly improves the physical adhesion of the electrodes, whereas those fabricated using a conventional dispensing printing technique peel off with a silver paste containing 2 wt% of a fluorosurfactant. Moreover, the contact resistivity and dimensionless deviation of total resistance are significantly reduced from 2.19 ± 1.53 mΩ·cm^2^ to 0.98 ± 0.92 mΩ·cm^2^ and from 0.10 to 0.03, respectively. By utilizing electrodes with an abnormally high-aspect-ratio of 0.79 (the measured thickness and width are 30.4 μm and 38.3 μm, respectively), the cell efficiency is 17.2% on a polycrystalline silicon solar cell with an emitter sheet resistance of 60 Ω/sq. This cell efficiency is considerably higher than previously reported values obtained using a conventional electrohydrodynamic jet printing technique, by +0.48–3.5%p.

Because a low-cost fabrication method is required in electronics industries to replace expensive photolithographic processes, numerous studies have been conducted using diverse printing techniques such as inkjet printing^1^,^2^,^3^, electrohydrodynamic jet printing^4^,^5^,^6^, aerosol jet printing^7^, and roll-to-roll printing^8^,^9^. However, previous studies have been primarily dedicated to the construction of fine, thin electrodes on a smooth surface because of the dominance of thin-film electronic devices. Meanwhile, there has been a clear and imminent industrial demand for fine electrodes with abnormally high aspect ratios on a rough surface in applications such as crystalline silicon solar cells. To reduce shading losses, the electrodes on the front surface of a crystalline silicon solar cell must be as fine as possible; while also being as thick as possible to minimize power loss. Moreover, high-aspect-ratio electrodes must be formed on the textured surface of a crystalline silicon solar cell with good adhesion and contact resistivity.

Because crystalline silicon solar cell wafers have become thinner and more vulnerable to breakage^10^, non-contact printing techniques such as inkjet printing, aerosol jet printing, electrohydrodynamic jet printing, and dispensing printing have been considered more suitable for the next-generation metallization of crystalline silicon solar cells in lieu of conventional contact printing techniques such as screen printing and stencil printing^11^.

Inkjet and aerosol jet printing techniques, which use the superimposition of acoustic waves generated from a piezoelectric actuator to eject a droplet out of a nozzle^12^ and the focused jet stream of a nebulized silver ink or paste through a nozzle^7^ (Fig. 1a,b), respectively, can be utilized to construct electrodes with a width of a few tens of micrometres. However, electrodes with aspect ratios above 0.5 are not readily obtainable by single-pass printing; thus, such electrodes require either multiple printing passes^13^ or subsequent metallization such as light-induced plating to thicken the electrodes^14^. Although light-induced plating is beneficial for adopting cheap metals such as copper for subsequent metallization, the isotropic deposition of a plating metal on the electrodes both thickens and widens the electrodes. Therefore, the benefit of subsequent metallization is compromised.

An electrohydrodynamic jet printing technique has drawn attention from those aiming to construct ultrafine electrodes with a width of a couple micrometres. By imposing a high electric field between the nozzle and the substrate^4^,^5^, as shown in Fig. 1c, the meniscus of the silver paste is pulled out to form a conical shape, *i.e*., a Taylor cone, because of the electrostatic force on the accumulated charges of the meniscus. The formation of a Taylor cone enables the construction of ultrafine electrodes. Because the viscosity of silver paste required for the stable formation of a Taylor cone is 2200–4200 cPs at a shear strain rate of 100 s^–1^ and a temperature of 23 °C, the electrohydrodynamic jet printing technique can be performed using a silver paste with a solid content as high as 85 wt%^15^, which is greater than the solid content of commercially available silver ink for an inkjet printing technique by +20 wt% p (NPS-J; solid content: 65 wt%; viscosity: 9 cPs; Harima Chemicals Group, Inc., Japan). However, the silver paste in this viscosity range still lacks yield stress, which plays a crucial role in preventing the as-printed electrodes from spreading and collapsing. Therefore, the construction of electrodes with high aspect ratios requires a stack of thin electrodes with multiple printing passes like inkjet and aerosol jet printing techniques^15^,^16^, which compromises the benefit of the electrohydrodynamic jet printing technique.

As an alternative to the aforementioned non-contact printing techniques, a dispensing printing technique, as shown in Fig. 1d, has been explored^17^,^18^. The capability to use a highly viscous silver paste with high yield stress makes the dispensing printing technique the most suitable technique for constructing high-aspect-ratio electrodes with single-pass printing^19^,^20^. Figure 2 exhibits the typical rheological behaviour of a shear thinning silver paste with yield stress in the course of dispensing printing. The viscosity of the silver paste rapidly decreases as the silver paste passes through the nozzle at a high shear strain rate, as shown in (A) of Fig. 2. After the silver paste is pushed out of the nozzle, the shear strain rate (except the extensional strain rate) is no longer applied to the silver paste; thus, its viscosity increases with time, as shown in (B). When the yield stress is recovered at a zero strain rate, as shown in (C), the silver paste finally stops spreading and holds its shape. Therefore, the rapid recovery in yield stress is necessary for the dispensable silver paste to construct fine electrodes with high aspect ratios.

Nonetheless, the high yield stress of the silver paste may lead to a poor contact, particularly at the interface between the silver paste and the textured surface of a crystalline silicon solar cell wafer, as shown in Fig. 3a. The silver paste with recovered yield stress stops wetting the textured surface, which consequently degenerates the adhesion and contact resistivity of the electrodes. Therefore, a new printing technique, *i.e*., electrostatic-force-assisted dispensing printing, is proposed here to produce fine and high-aspect-ratio electrodes with improved adhesion and contact resistivity.

## Results and Discussion

**Figure 3:**
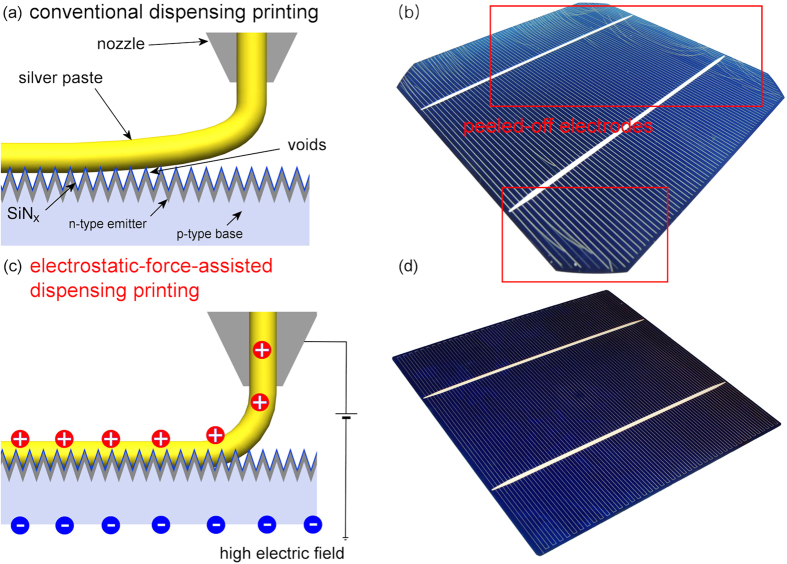
Schematic illustration of the interfacial contact of the silver paste on the textured surface of a crystalline silicon solar cell, which was fabricated using a silver paste with 2 wt% fluorosurfactant and a dispensing printing technique in (a–b) or an electrostatic-force-assisted dispensing printing technique in (c–d).

The electrostatic-force-assisted dispensing printing technique has the characteristics of both the dispensing and electrohydrodynamic jet printing techniques. As a dispensing printing technique, this approach enables the usage of a highly viscous silver paste with high yield stress. As an electrohydrodynamic jet printing technique, it applies an electric field between the nozzle and the crystalline silicon solar cell wafer, as shown in Fig. 3c. To compare the electrostatic-force-assisted dispensing printing technique with a conventional dispensing printing technique, electrodes were constructed on crystalline silicon solar cell wafers using both printing techniques with an in-house developed silver paste containing 2 wt% of a fluorosurfactant. This surfactant caused the silver paste to have poor adhesion properties.

As shown in Fig. 3b, the dispensing printed electrodes peeled off from the textured surface of the crystalline silicon solar cell wafer after co-firing because the fluorosurfactant decreased the surface energy of the textured crystalline silicon solar cell wafer during co-firing and hampered the adhesion of the electrodes to the textured surface. In contrast, the electrodes that were constructed with the electrostatic-force-assisted dispensing printing technique remained firmly adhered to the textured surface of the crystalline silicon solar cell wafer, as shown in Fig. 3d.

It is noteworthy that the viscosity of the in-house developed silver paste was above 20000 cPs at a shear strain rate of 100 s^−1^ and a temperature of 23 °C, as shown in Supplementary Fig. S1, so that no Taylor cone could be formed by the electrohydrodynamic jet printing mode. Instead, the syringe pump in the eNano printer was directly used to force the in-house developed silver paste to be dispensed out of the nozzle. Supplementary Figure S2 clearly shows that the dispensed silver paste was attracted to the textured surface of the crystalline silicon solar cell wafer when a voltage of 0.7 kV was applied. It indicates the electrostatic force, or the Coulomb force, rather than electrowetting is the primary reason of the enhanced adhesion of silver paste to the textured surface of the crystalline silicon solar cell wafer.

**Figure 4:**
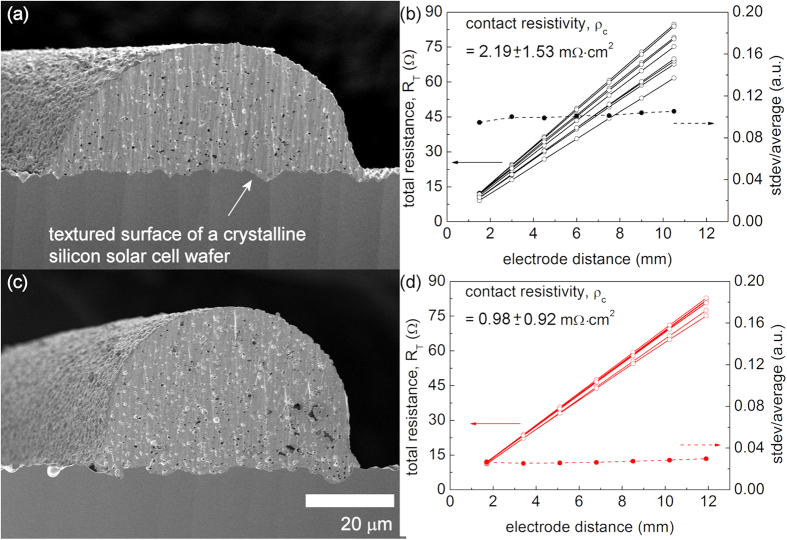
Cross-sectional SEM images and plots of the total resistance (R_T_) as a function of the electrode distance. The electrodes were fabricated using a dispensing printing technique (0 wt% fluorosurfactant) in (**a**–**b**) and an electrostatic-force-assisted dispensing printing technique (2 wt% fluorosurfactant) in (**c**–**d**).

Crystalline silicon solar cells were produced using two methods: a dispensing printing technique with a silver paste that did not contain a fluorosurfactant and an electrostatic-force-assisted dispensing printing technique with a silver paste that contained 2 wt% of a fluorosurfactant. The cross-sectional morphologies and electrical properties of the solar cells were compared. As shown in Fig. 4a,c, the interfacial contact of the electrode constructed using the electrostatic-force-assisted dispensing printing technique with a silver paste containing 2 wt% of a fluorosurfactant exhibited no notable difference from that of the electrode constructed using the dispensing printing technique with silver paste without a fluorosurfactant. Moreover, the contact resistivity of the electrodes constructed using the electrostatic-force-assisted dispensing printing technique was 0.98 ± 0.92 mΩ·cm^2^, which is much lower than that of the electrodes constructed using the dispensing printing technique (2.19 ± 1.53 mΩ·cm^2^). The dimensionless deviation of total resistance is defined here as the standard deviation divided by the average total resistance at each electrode distance. For the electrostatic-force-assisted dispensing printing technique, this value is 0.03, which is much lower than that for the dispensing printing technique (0.10).

To compare the electrostatic-force-assisted dispensing printing technique with the conventional electrohydrodynamic jet printing technique, polycrystalline silicon solar cells (nominal emitter sheet resistance: 60 Ω/sq; Hanwha Chemical R&D Centre, Republic of Korea) were fabricated with the in-house developed silver paste, which contained 2 wt% of a fluorosurfactant, using the electrostatic-force-assisted dispensing printing technique. In addition, solar cells were fabricated using either a diluted version of a commercially available silver paste or a second in-house developed silver paste that did not contain a fluorosurfactant, using the conventional electrohydrodynamic jet printing technique (the detailed recipe for formulating the second in-house developed silver paste is described in ref. 15).

**Figure 5:**
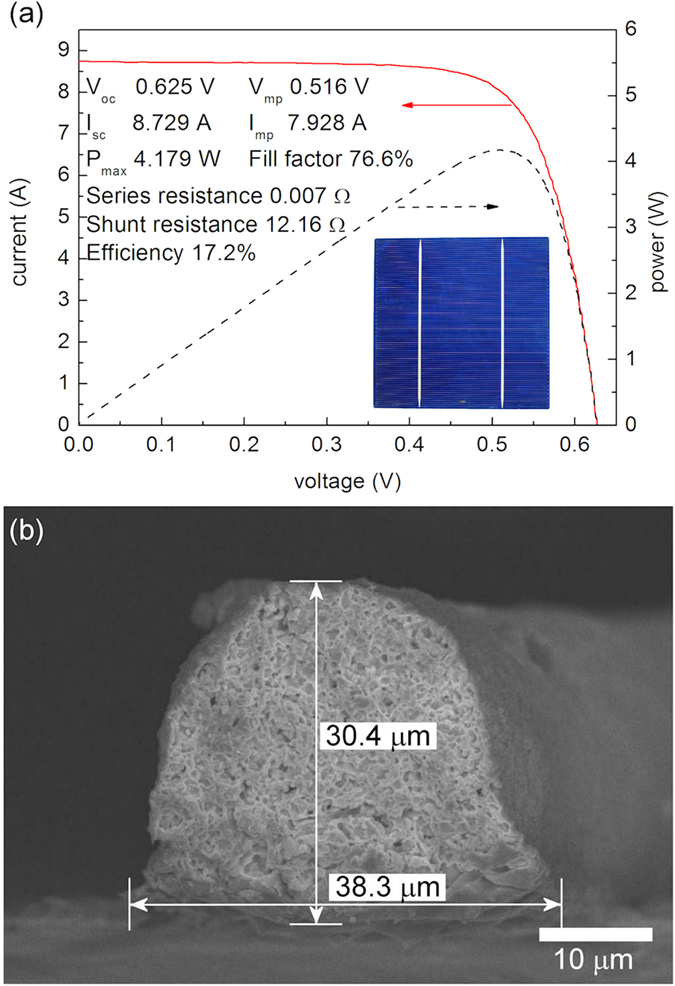
I–V curve of a polycrystalline silicon solar cell fabricated using the electrostatic-force-assisted dispensing printing technique and a silver paste with 2 wt% of a fluorosurfactant (a); cross-sectional SEM image of its electrode (b).

The highest cell efficiency for the polycrystalline silicon solar cell fabricated using the electrostatic-force-assisted dispensing printing technique with single-pass printing was 17.20%, even for the in-house developed silver paste containing 2 wt% of a fluorosurfactant, as shown in Fig. 5a. This efficiency is considerably higher than those for the cells that were fabricated using the conventional electrohydrodynamic jet printing technique: 13.7% for 20 printing passes with the diluted version of a commercially available silver paste^16^ and 16.72% for 30 printing passes with the second in-house developed silver paste without a fluorosurfactant^15^. The cross-sectional SEM image in Fig. 5b also shows the aspect ratio of the electrode constructed using the electrostatic-force-assisted dispensing printing technique, which was as high as 0.79; the measured thickness and width were 30.4 μm and 38.3 μm, respectively.

## Conclusion

We herein report a novel printing technique that can overcome the limitations of conventional dispensing and electrohydrodynamic jet printing techniques. Although the electrohydrodynamic jet printing technique can be utilized to construct ultrafine electrodes, multiple printing passes are inevitably required to construct high-aspect-ratio electrodes. A dispensing printing technique is more beneficial for constructing high-aspect-ratio electrodes using single-pass printing with a highly viscous silver paste with high yield stress. However, the high yield stress of the silver paste results in a poor contact at the interface between the silver paste and the textured surface of the crystalline silicon solar cell wafer; hence, the high yield stress degenerates the adhesion and contact resistivity. The electrostatic-force-assisted dispensing printing technique, which has the characteristics of both the dispensing and electrohydrodynamic jet printing techniques, can be employed to construct abnormally high-aspect-ratio electrodes using single-pass printing and results in excellent physical adhesion and reduced contact resistivity on a rough surface. These unique traits of the electrostatic-force-assisted dispensing printing technique render this approach the best fit for the next-generation metallization of crystalline silicon solar cells.

## Methods

### Preparation of the in-house developed silver paste

The primary inorganic ingredients were silver powder (HP-0710, Heesung Metal Ltd., Republic of Korea), glass frit (V2172, Ceradyne Inc., USA) and zinc oxide nanopowder (Order No. 544906, Sigma-Aldrich Corp., USA) at a weight ratio of 90:5:5, respectively. The total solid content of the inorganic ingredients in the in-house developed silver paste was 88 wt%. The solid contents of the organic ingredients with respect to the total weight of the inorganic ingredients were: 1 wt% of ethyl cellulose (Order No. 200646, Sigma-Aldrich Corp., USA) as an organic binder, 1 wt% of polyamide thixotrope (Elementis Specialties, Inc., USA) as a rheological modifier, and α-terpineol (CAS No. 98-55-5, Kanto Chemical co., Inc., Japan) as a carrier vehicle. In addition, 2 wt% of a fluorosurfactant (Novec^TM^ Fluorosurfactant FC-4430, Energy and Advanced Materials Division, 3M, USA) with respect to the total weight of inorganic ingredients was added to obtain poor adhesion properties. The viscosity of the in-house developed silver paste was measured with a rheometer (RheoScope 1, Thermo Fisher Scientific Inc., Germany).

### Fabrication of crystalline silicon solar cells

Polycrystalline silicon solar cell wafers with a nominal emitter sheet resistance of 60 Ω/sq (Hanwha Chemical R&D Centre, Republic of Korea) were used for measuring the cell efficiency. After an aluminium paste was screen-printed on the rear side of a polycrystalline silicon solar cell wafer, it was dried at 180 °C for 7 min on a hot plate (DHSL.HP2020300, DHSL Korea Co., Ltd., Republic of Korea). Then, two busbars on the front side of the polycrystalline silicon solar cell wafer were screen-printed with a commercially available silver paste and dried on a hot plate at 150 °C for 3 min. To demonstrate the unique characteristics of the electrostatic-force-assisted dispensing printing technique, a standard version of the eNano printer (Enjet Co., Republic of Korea) was used, with a 40-μm-diameter ceramic nozzle. A printing speed of 7 mm/s was used, and a voltage of 0.7 kV was applied for a standoff distance of less than 100 μm between the nozzle and the polycrystalline silicon solar cell wafer. For comparison, dispensing printing was also conducted using a dispensing robot (EzROBO-5 GX ST2520, Iwashita Engineering, Inc., Japan) with a 75-μm-diameter ceramic nozzle. The printing speed was 100 mm/s, which was considerably higher than the printing speed of the eNano printer due to a high dispensing capability with a powerful screw pumping motor (AMD3-CEC-YD 100 W, Eser Corp., Japan), and the standoff distance was 1 mm. The electrode spacing was set to be 1.5 mm or 1.7 mm. The as-printed electrodes were dried at 150 °C for 3 min on a hot plate (DHSL.HP2020300, DHSL Korea Co., Ltd., Republic of Korea) and subsequently co-fired at a peak temperature of 820 °C using a rapid thermal processor (AccuThermo AW 610, Allwin21 Corp., USA). Finally, edge isolation was performed using a 532-nm laser (NANIO 532-10-V, InnoLas Laser GmbH, Germany) and a 2-axis laser scanner (SS-II-10, Raylase AG, Germany).

### Electrical and morphological characterization

The electrical properties were evaluated using a probe station (MST 4000 A, MS Tech Co., Ltd., Republic of Korea) and a source meter (Model 2401, Keithley Instruments Inc., USA). For morphological characterization of the electrodes, a field-emission scanning electron microscope (SUPRA-55VP, Carl Zeiss NTS GmbH, Germany) was used.

## Additional Information

**How to cite this article**: Shin, D.-Y. *et al*. Electrostatic-Force-Assisted Dispensing Printing to Construct High-Aspect-Ratio of 0.79 Electrodes on a Textured Surface with Improved Adhesion and Contact Resistivity. *Sci. Rep*. **5**, 16704; doi: 10.1038/srep16704 (2015).

## Supplementary Material

Supplementary Information

## Figures and Tables

**Figure 1:**
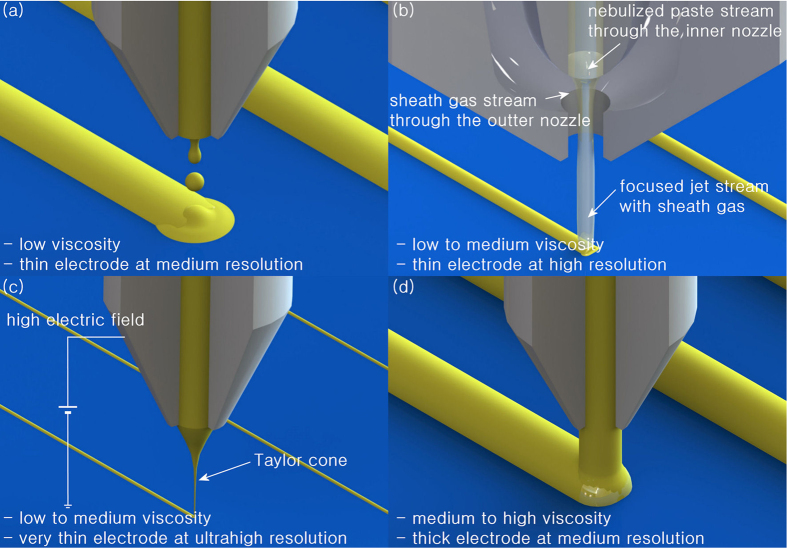
Schematic illustration of various non-contact printing techniques: (a) inkjet printing, (b) aerosol jet printing, (c) electrohydrodynamic jet printing, and (d) dispensing printing.

**Figure 2:**
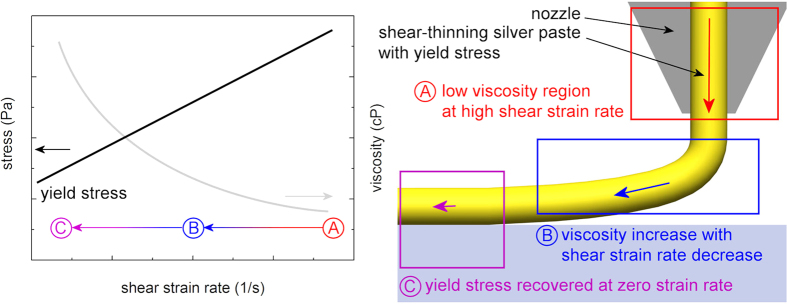
Schematic illustration of the rheological behaviour of the shear thinning silver paste with yield stress in the course of dispensing printing.
