# Supplementary material for: Electrostatic-Force-Assisted Dispensing Printing to Construct High-Aspect-Ratio of 0.79 Electrodes on a Textured Surface with Improved Adhesion and Contact Resistivity
Source: Sci Rep. 2015 Nov 18;5:16704. doi: 10.1038/srep16704 (PMC4649362; doi:10.1038/srep16704)
Supplement: Supplementary Information [file srep16704-s1.pdf]

Electrostatic-Force-Assisted Dispensing Printing to Construct High-Aspect-Ratio of 0.79 Electrodes on a Textured Surface with Improved Adhesion and Contact Resistivity

Dong-Youn Shin[1,*], Sung-Soo Yoo[1], Hee-eun Song[2,*], Hyowon Tak[3] & Doyoung Byun[4]

[1]Department of Graphic Arts Information Engineering, Pukyong National University, 365, Sinseon-ro, Nam-gu, Busan, 608-739, Republic of Korea. [2]Solar Energy Research Centre, Korea Institute of Energy Research, Daejeon, 305-343, Republic of Korea. [3]Enjet Inc., Ltd., 2066, Seobu-ro, Jangan-gu, Suwon-si, Gyeonggi-do,440-746, Republic of Korea. [4]Department of Mechanical Engineering, Sungkyunkwan University, 2066, Seobu-ro, Jangan-gu, Suwon-si, Gyeonggi-do,440-746, Republic of Korea. Correspondence and requests for materials should be addressed to D.-Y. S. (email: dongyoun.shin@gmail.com, Tel: +82-51-629-6394) or H.-E. S. (email: hsong@kier.re.kr)

Supplementary information

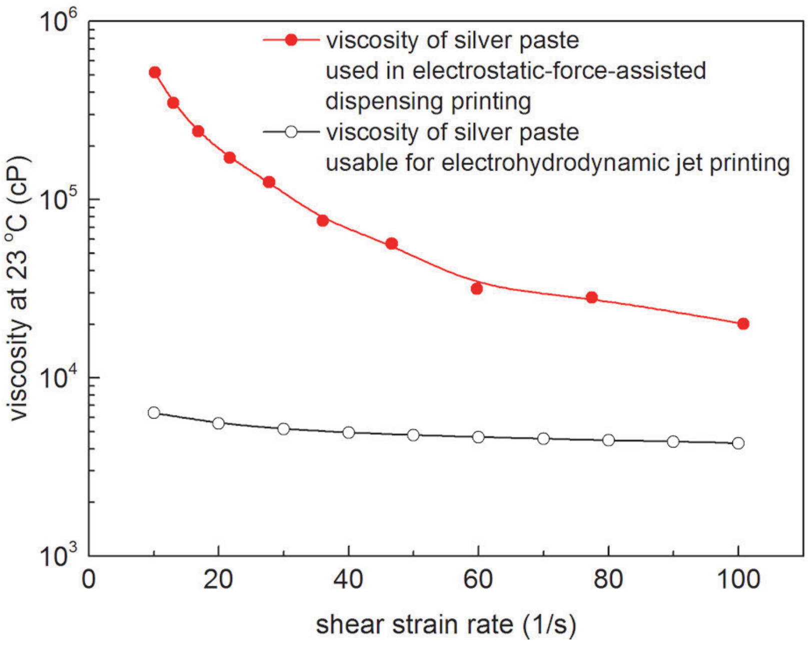

Supplementary Figure S1. Viscosity comparison of the in-house developed silver paste and silver paste for electrohydrodynamic jet printing.

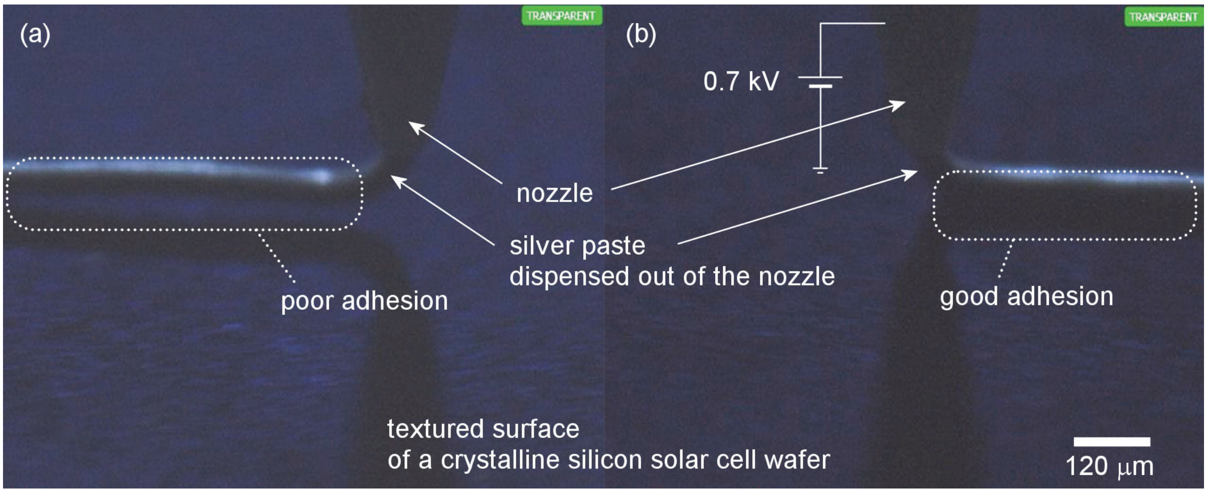

Supplementary Figure S2. Comparison of the adhesion behaviours of dispensed silver paste, (a) without and (b) with a voltage of 0.7 kV.
